# Comprehensive Review of Polysaccharide-Based Materials in Edible Packaging: A Sustainable Approach

**DOI:** 10.3390/foods10081845

**Published:** 2021-08-10

**Authors:** Yuan Zhao, Bo Li, Cuicui Li, Yangfan Xu, Yi Luo, Dongwu Liang, Chongxing Huang

**Affiliations:** 1 School of Light Industry & Food Engineering, Guangxi University, 100 Daxue Road, Nanning 530004, China; zy199113@163.com (Y.Z.); 13654529477@163.com (B.L.); licuicui081@163.com (C.L.); xyflower23@163.com (Y.X.); ly956662@163.com (Y.L.); huangcx@gxu.edu.cn (C.H.); 2 Guangxi Key Laboratory of Clean Pulp & Papermaking and Pollution Control, College of Light Industry and Food Engineering, Guangxi University, Nanning 530004, China; 3 Key Laboratory of Processing Suitability and Quality Control of the Special Tropical Crops of Hainan Province, Wanning 571533, China

**Keywords:** polysaccharide-based materials, edible packaging, cellulose, hemicellulose, starch, chitosan, polysaccharide gums

## Abstract

Edible packaging is a sustainable product and technology that uses one kind of “food” (an edible material) to package another kind of food (a packaged product), and organically integrates food with packaging through ingenious material design. Polysaccharides are a reliable source of edible packaging materials with excellent renewable, biodegradable, and biocompatible properties, as well as antioxidant and antimicrobial activities. Using polysaccharide-based materials effectively reduces the dependence on petroleum resources, decreases the carbon footprint of the “product-packaging” system, and provides a “zero-emission” scheme. To date, they have been commercialized and developed rapidly in the food (e.g., fruits and vegetables, meat, nuts, confectioneries, and delicatessens, etc.) packaging industry. However, compared with petroleum-based polymers and plastics, polysaccharides still have limitations in film-forming, mechanical, barrier, and protective properties. Therefore, they need to be improved by reasonable material modifications (chemical or physical modification). This article comprehensively reviews recent research advances, hot issues, and trends of polysaccharide-based materials in edible packaging. Emphasis is given to fundamental compositions and properties, functional modifications, food-packaging applications, and safety risk assessment of polysaccharides (including cellulose, hemicellulose, starch, chitosan, and polysaccharide gums). Therefore, to provide a reference for the development of modern edible packaging.

## 1. Introduction

Since the 19th century, petroleum-based polymers and plastics have occupied a major position in food packaging, but most are non-renewable, non-biodegradable, difficult to recycle, and carelessly discarded as garbage after use, thereby contributing to ecological environmental deterioration and possible health hazards [1]. Under various natural and anthropogenic forces, plastic fragments (from waste plastic containers, sheets, and films) break down into small particle sizes, further generating microplastics with a diameter smaller than 5 mm [1,2,3]. According to Lebreton et al. [4], over 79,000 tons of plastic waste float on the Great Pacific Garbage Patch, and the content of marine microplastics has increased rapidly from 0.4 kg/km^2^ in the 1970s to 1.23 kg/km^2^ in 2015. Then Barrett et al. [5] estimated that there could be as much as 14.4 million tonnes of microplastics in the top 9 cm of sediment throughout the global ocean, which was 34–57 times more than that at the ocean surface. Moreover, microplastics have been ubiquitously detected in oceans (from the continental shelf to deep-sea waters [6], from the eastern North Pacific Ocean [3] to the Indian Ocean [7], and from coral reef to whales [8]), freshwater systems [9], airborne [10], plants, animals, and even humans [11,12]. Unfortunately, the plastic (including microplastics) pollution is posing a serious threat to the global environment and human health. Therefore, it is of great significance for packaging to develop a series of renewable environment-friendly materials to replace the traditional petrochemical-based materials, among which the edible material is one of the most promising materials.

**Figure 1:**
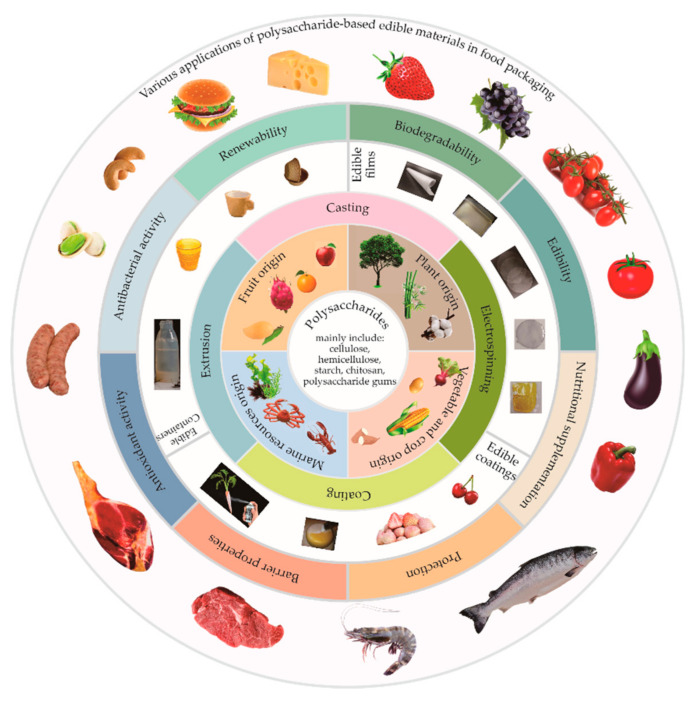
The major sources, types, processing methods, product forms, and food preservation applications of polysaccharide-based edible packaging.

Edible packaging material is a kind of sustainable material that takes natural, edible and digestible “food” as raw material and is processed by modern material forming technology. It has excellent biocompatibility and biodegradability and can be consumed by animals or humans along with the food, while satisfying the basic functions of packaging (e.g., protection and transport), thus avoiding packaging waste pollution [13,14]. The design of edible packaging was originally inspired by the “peel/skin” of fruits and vegetables, and now edible packaging has been widely applied to various forms of food packaging (e.g., films, coatings, sheets, bags, cups, trays, and lids), as shown in Figure 1. In addition, edible packaging materials are non-toxic harmless, can be in direct contact with food, and even can be used as carriers of some antioxidative, antibacterial and/or nutritional factors to improve the sensory quality and nutritional value of foods [14,15].

To date, edible packaging materials include three natural biopolymers: polysaccharides, proteins, and lipids, among which polysaccharides (the most abundant natural macromolecules in nature, low processing cost and special function) occupy the most important position [13]. Polysaccharides are complex carbohydrates with varying degrees of polymerization and are composed of monosaccharides linked by α-1,4-, β-1,4-, or α-1,6-glycosidic bonds [16]. The polysaccharides commonly applied in edible packaging are cellulose, hemicellulose, starch, chitosan, and polysaccharide gums, which are used as the main matrix of packaging materials, and processed into polysaccharide-based edible films or layers by casting, coating, electrospinning, or extrusion technologies (Figure 1) [15,16,17,18].

The main value of polysaccharide-based edible packaging materials is to protect the quality of food, prolong their shelf life, and improve the functional characteristics, economic benefits, and sustainability of the packaging [15,19]. Compared with traditional packaging materials (such as paper, plastic, metal, and glass), polysaccharide-based materials have two significant advantages: Edibility and environmentally friendly performance. Compared with protein- and lipid-based packaging materials, polysaccharides have better chemical stability and processing adaptability, a greater range of sources, and lower cost. According to relevant studies, polysaccharide-based materials have good gases, aromas, and lipids barrier properties [20,21,22,23,24]; and even some polysaccharides and their derivatives have antioxidant and antimicrobial activities, which can effectively protect foods (e.g., fruits, vegetables, meat, aquatic products, nuts, confectioneries, and delicatessens), and extend their shelf life [15,19]. Furthermore, developing polysaccharide-based materials effectively reduces the dependence on petroleum resources, decreases the carbon footprint of the “product-packaging” system, and meets the strategic requirements of global sustainable packaging.

This article reviews the latest advances in the major polysaccharide-based edible packaging materials (cellulose, hemicellulose, starch, chitosan, and polysaccharide gums) from the viewpoints of fundamental compositions, properties, functional modification, application, and safety, highlights the potential of polysaccharides in food packaging, and provides the trends of these materials in modern packaging technology.

## 2. Fundamental Compositions and Properties of Various Polysaccharides

**Figure 2:**
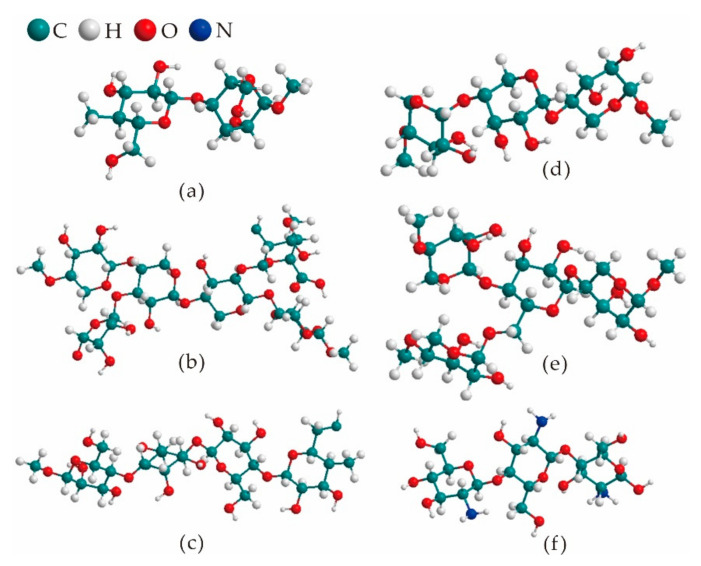
Three-dimensional models of the molecular structure of various polysaccharides. (**a**): Cellulose (**b**): Xylan (**c**): Glucomannan (**d**): Amylose (**e**): Amylopectin (**f**): Chitosan.

The functional characteristics of food packaging are not only related to the properties and main deterioration modes of packaged foods, but also depend on the compositions and properties of the packaging materials. Therefore, the relevant discussion of various polysaccharides has important guiding significance for analyzing the applicability of different polysaccharides in food packaging, as well as the selection of corresponding modification and application schemes. The major and minor sources, similarities and differences in compositions and structures of five polysaccharides, as well as their outstanding advantages as edible packaging are shown in Table 1. Meanwhile, the molecular structure models of different polysaccharides are shown in Figure 2.

Although the reported polysaccharides differ in source, composition, structure, and characteristics, they generally have good gelation, film-forming, mechanical, and barrier properties, and are abundant, renewable, edible and biodegradable. In particular, there are many kinds of hemicellulose and polysaccharide gums, but the ones commonly used in packaging are xylan, glucomannan, pectin, alginate, carrageenan, and agar. These polysaccharides can be processed into different forms of packaging (including films, coatings, containers, sponges, and gels) through various material technologies, and have tremendous potential in the development and application of edible packaging in the future.

However, compared with traditional petroleum-based polymers and plastics, polysaccharide-based materials still have many disadvantages, mainly including the following:

(1) The chemical and thermal stability of polysaccharides are poor, which is not conducive to their subsequent molding processing. In particular, the materials formed by only one kind of polysaccharide are often brittle, easy to crack or wrinkle, have high shrinkage after molding, and have poor mechanical properties.
(2) Polysaccharide-based materials contain many hydroxyl, amino or carboxyl groups, which result in high hydrophilicity, easy swelling by moisture, and poor water vapor barrier and moisture resistance. Moreover, they are sensitive to water, and their hydrogen bonding actions, microstructures and internal stress would change after moisture absorption; which resulted in a significant decrease in the mechanical strength of polysaccharide-based materials at high relative humidity [81,82,83].
(3) Cellulose, hemicellulose, starch, agar, and other polysaccharides (except chitosan, pectin, and their derivatives) would provide nutrients and facilitate the growth and reproduction of microorganisms, which is not conducive to food storage.

## 3. Modifications of Various Polysaccharide-Based Materials for Edible Packaging

Given the above limitations, polysaccharide-based materials should be modified based on the actual application requirements to optimize their functional properties and promote their application in edible packaging. The existence of functional groups such as hydroxyl, amino, acetylamino, and carboxyl groups in polysaccharides creates conditions for their material modification. Currently, the commonly used modification techniques are chemical and physical modifications.

### 3.1. Chemical Modifications of Polysaccharide-Based Materials

Common methods of polysaccharide chemical modification include functional group modification, graft copolymerization, and cross-linking (Table 2). Functional group modification refers to the modification of some functional groups on the main chain and/or side chain of polysaccharides to obtain modified polysaccharides with improved physical and chemical properties through etherification (e.g., carboxymethylation and hydroxypropylation), esterification (e.g., organic acid and anhydride esterification), quaternization, and acylation [32,84,85,86]. Graft copolymerization refers to the process by which the polysaccharide active groups (e.g., hydroxyl, amino, and carboxyl) react with other monomers to obtain target polysaccharides under the action of an initiator or radiation [32,87]. Cross-linking refers to the process in which polysaccharides are polymerized within themselves or with macromolecules of other materials under the action of cross-linking agents (which can improve the cross-linking degree between substances) to obtain cross-linked polysaccharides with a network structure, thus enhancing the stability and physical properties of polysaccharides [85]. For example, polysaccharides are linked with proteins (whose mechanical properties are often better than polysaccharides) to obtain polysaccharide-protein complexes with optimized properties based on reducing the electrostatic free energy of the system by electrostatic interaction. In particular, during cross-linking, the thermal and mechanical properties of polysaccharide-based materials can be further improved by using carboxylic acid or calcium ions as cross-linking agents [88].

For cellulose, the goal of chemical modification is to reduce the hydrogen bond strength and improve the processing adaptability of the materials. Various properties of cellulose-based materials (e.g., permeability, solubility, mechanical properties, barrier properties, and thermoplastic behavior) can be adjusted by changing the degree of substitution, type of chemicals, and polymer chain length [114]. Methylation, carboxymethylation, hydroxypropylation, and acetic acid esterification are often used to replace the hydroxyl groups of cellulose (Table 2). For instance, the mechanical and water vapor barrier properties of edible films, prepared using modified hydroxypropyl methylcellulose (HPMC), obtained by increasing the degree of hydroxyl substitution and relative molecular weight, were significantly improved [83]. The modified methylcellulose (MC) has high solubility and efficient oxygen and lipid barrier properties. A water-soluble edible packaging bag made of MC/HPMC composites has better mechanical and barrier properties, which are suitable for packaging dry food ingredients [81]. Furthermore, compared with other polymers in the previous literature, the tensile strength of MC films (15.78 MPa) was better than that of collagen and whey protein films [91], and even was higher than that of low-density polyethylene films (0.9–14 MPa) and poly(ε-caprolactone) (14 MPa). Moreover, the corresponding elongation at break (15.4%) was superior to polystyrene (2–3%), poly (3-hydroxybutyrate) (5–8%), and poly(L-lactic acid) (9%) [115,116]. Besides, the cross-linking [90] and graft copolymerization [100] could give cellulose-based materials better surface morphology and mechanical properties, even light resistance, antioxidant, and/or antimicrobial activities.

Chemical modification of hemicellulose is often conducted through carboxymethylation, hydroxypropylation, esterification, acetylation, and cross-linking (Table 2) [32,87,101,102]. Ramos et al. [101] prepared two kinds of functional xylans, carboxymethyl xylan (CMX) and 2-dodecenyl succinic anhydride-modified xylan (X-2-DSA), using beech xylan as the raw material, and then prepared different films. The results showed that X-2-DSA film possessed similar tensile strength and oxygen permeability to CMX film. Whereas the elongation at break of X-2-DSA film was almost 3.75 times that of the latter one, and the water vapor permeability of CMX film was about 2.3 times that of the former. These phenomena might be due to the replacement of some hydroxyl groups by non-polar long aliphatic carbon chains of dodecenyl succinic anhydride, which obtains plasticizing effect and makes the xylan less polar. Additionally, Mikkone et al. [87] modified xylan by hydroxypropylation (playing an internal plasticization role) and sorbitol was added as an external plasticizer to prepare a xylan-based barrier film via the casting method. The results indicated that the combination of xylan and sorbitol with a certain degree of hydroxypropyl substitution (from low to medium is 0.3 to 1.1) improved the film formability, flexibility, thermal stability, and barrier properties of the composite films. In particular, the composite film with the lowest hydroxypropyl substitution degree (0.3) had the best comprehensive properties (e.g., the highest tensile strength and the lowest oxygen and water vapor permeabilities), and the best biomass use and biodegradability.

The objective of chemical modification of the original starch is to reduce its moisture absorption and water sensitivity, heighten the compatibility of starch with other hydrophobic materials, and improve its processing adaptability [117]. Therefore, researchers often use highly hydrophobic groups to replace hydrophilic -OH groups through chemical modification methods, such as carboxymethylation, acetylation, esterification [84,106], polymer grafting, cross-linking, and “click chemistry”, which reduce the polarity of starch-based materials and improve their mechanical properties (Table 2). Liu et al. [118] first prepared carboxylated starch (which has higher hydrophilicity and polarity than that of native starch, but lower gelatinization temperature and enthalpy) by bio-α-amylase catalysis, and then introduced CMC into the modified starch matrix to enhance the hydrophobicity, thermal stability and mechanical strength of starch-based materials. In particular, the tensile strength of carboxylated starch composite films reached a maximum value of 44.8 MPa at 15% CMC addition, the hydrophobic property was effectively improved when CMC > 10%, and the static water contact angle was 66.8° at 35% CMC addition. Similarly, other researchers have modified starch by chemical methods first, but then combined it with the unmodified natural starch to produce better composites [17,119,120]. Notably, the FDA has limitations on the reagents and reactions, which are used for the manufacturing of food-grade modified starch [46], so we should follow the applicable regulations and standards when preparing starch-based edible packaging, as well as other polysaccharides edible packaging.

The purpose of the chemical modification of chitosan is to increase its water solubility, thermal stability, mechanical properties, barrier properties, and antibacterial activity, and the main chemical methods include carboxymethylation, acylation, quaternization, graft copolymerization, and cross-linking (Table 2) [58,88,121]. For example, carboxymethyl chitosan was formed by introducing carboxymethyl into N or O atoms of the chitosan skeleton through reactions of halogenated acetic acid or glyoxylic acid, thus enhancing the water solubility and adhesion [58]. This modification could also improve the antibacterial properties, with a wide range of carboxymethylation degrees. *O*-carboxymethyl and *N*,*O*-carboxymethyl chitosans showed better antibacterial activity than ordinary chitosan, and with the increase in carboxymethylation, the antibacterial activity of *O*-carboxymethyl chitosan increased first, then decreased [122]. Likewise, the water solubility and antimicrobial activity of the original chitosan also improved by grafting glycidyltrimethylammonium chloride [123] or nisin [124] onto the chitosan chain. Furthermore, Li et al. [125] introduced monophenol and ortho-diphenol to chitosan to obtain functionalized chitosan derivatives owned high antioxidant activity, which the EC_50_ of inhibition of DPPH, hydroxyl (·OH), and superoxide (O_2_·-) radical-scavenging was 0.041–0.172, 0.010–0.089, and 0.014–0.038 mg/mL, respectively. Tan et al. [126] synthesized amino- and acylhydrazine-functionalized chitosan derivatives via 1,2,3-triazole and 1,2,3-triazolium by Cuprous-catalyzed azide-alkyne cycloaddition and *N*-methylation, which displayed stronger antioxidant capacity (especially against superoxide anion radical) than pristine and hydroxyl-modified chitosan. Besides, *N*-methylation of 1,2,3-triazoles further strengthened their antioxidant action. These chitosan derivatives had no cytotoxicity on L929 (at 0.0625 mg/mL) or HaCaT (at 0.1 mg/mL) cells, showing bright prospect in novel antioxidant edible packaging. In addition, the reaction of amino and hydroxyl groups in chitosan with polyaldehydes, polyesters, or polyethers can lead to cross-linking in the composite system, forming a three-dimensional network structure, thus, enhancing the thermal stability, mechanical properties, and barrier properties of chitosan [33,88]. Notably, the introduction of a cross-linking agent can further improve the properties of chitosan-based materials [33,127]. Chen et al. [33] added fulvic acid as a cross-linking agent to a konjac glucomannan/chitosan matrix to improve the thermostability, optical properties, and tensile strength (57.79 MPa, increased by 41.16%) of the composite film, while reduced its WVP (as low as 5.25 g·Pa^−1^·s^−1^·m^−1^, decreased by 39.31%).

In addition, the chemical modifications of polysaccharide gums (e.g., pectin, alginate, carrageenan, and agar) are mainly carboxymethylation, hydroxylation, acylation, esterification, graft copolymerization, and cross-linking (Table 2) [86,88,113,128]. For instance, Cao et al. [112] modified the original agar via carboxymethylation, while decreasing the dissolving temperature, gelling temperature, gel strength, hardness, fragility, adhesiveness, gumminess, and chewiness of carboxymethyl agar (CMA) by increasing carboxymethyl groups, conversely improving the springiness and cohesiveness of CMA, and enhancing the compactness of CMA skeleton structures. Based on polysaccharide gums and carboxymethyl cellulose being rich in active groups (-COOH and -OH) and have polyanion properties, Šešlija et al. [113] modified pectin with carboxymethyl cellulose and added glycerol and calcium chloride (which promote cross-linking through calcium ions), thus improving the thermal stability and mechanical strength of the composite film.

### 3.2. Physical Modifications of Polysaccharide-Based Materials

The most common and simple method for physical modification of polysaccharides is blending, namely blending one kind of polysaccharide with another or more edible materials (e.g., another polysaccharide, protein, and lipid), while supplementing with edible plasticizers, compatibilizers, antioxidants or antibacterial agents, and other small molecular additives (e.g., glycerin, essential oil, and other plant extracts). Therefore, complementary advantages of different materials are achieved while optimizing their comprehensive functions (Table 3) [81]. For example, proteins and polysaccharides are blended to form edible composites, in which positively charged proteins and anionic polysaccharides are attracted to each other to form highly structured compounds, and the water solubility, interfacial properties, adsorption, mechanical properties, and barrier properties of the composites are better than those of a single material [129,130]. Furthermore, when adding lipids into the polysaccharide/protein matrix, polysaccharides or proteins with high surface activity reduces the surface tension in the lipid emulsion, forms a space layer around the lipid droplets to enhance the emulsifying ability, promotes the stability of the emulsion, and ensure the mechanical strength and structural integrity of the composites. However, hydrophobic lipids reduce water migration and enhance the water resistance and water vapor barrier properties of the composites [131,132]. Overall, the water resistance, barrier properties, mechanical properties, heat sealing properties, and transparency of the polysaccharide-based composites could be further optimized, and even new functional activities could be developed by adjusting the composition and proportion of raw materials during blending. In general, the cohesion of a complex material increases with an increase in the length and polarity of the polymer chain, thus, improving the strength and abrasion resistance of its products, as well as the barrier properties to gas, water vapor, and solute. However, the enhancement of structural cohesion would lead to a decrease in the flexibility, porosity, and transparency of materials. Therefore, the types, proportions, and processing methods of raw materials should be explored according to the application requirements of polysaccharide-based edible packaging.

For cellulose, the functional properties of cellulose-based packaging materials can be further optimized through physical blending reinforcers, barrier factors, antioxidants, or antimicrobials into the cellulose matrix (Table 3). Esther et al. [154] significantly improved the antioxidant, antibacterial, and barrier properties of carboxymethyl cellulose-based edible films by adding concentrated bay leaf essential oil. The results showed that when the content of essential oil was 15% (*w*/*w*), compared with the unmodified carboxymethyl cellulose film, the antioxidant activity of the composite film was improved (as high as 99%), which slowed down lipid oxidation in food and effectively inhibited the growth of *Escherichia coli* and *Candida glabrata*, the water vapor barrier property was increased by 50%, and almost 100% ultraviolet light was blocked. Other studies have found that the antioxidant and antibacterial activities of cellulose-based materials can also be improved by adding dipalmitoyl lecithin liposomes (loaded with quercetin and rutin) [133], α-tocopherol [94], spent coffee grounds’ polysaccharides [95] and bacteriocin (from *Bacillus methylotrophicus* BM47) [27].

Functional hemicellulose-based edible materials can be obtained by the physical blending of hemicellulose with other polysaccharides, proteins, lipids, or other animal and plant extracts (Table 3) [127,135,136]. Along with konjac glucomannan (KGM), Wang et al. [135] improved the thermal stability, mechanical and water vapor barrier properties of KGM-based edible films by introducing microcrystalline cellulose loaded with polydopamine. Wu et al. [127] integrated chitosan/gallic acid nanoparticles with a KGM matrix to reduce the free volume of this blending system, significantly improving the mechanical and barrier properties of the edible composite film, while endowing the films with good antibacterial activity (for *Staphylococcus aureus* and *E. coli* O157:H7). Likewise, electrospun KGM/zein edible nanofiber films loaded with curcumin were prepared by Wang et al. [136] for application in food packaging. The addition of zein caused an increase in the thermal properties and hydrophobicity based on the interactions of hydrogen bonds between KGM and zein, whereas curcumin functioned as an antioxidant [2,2-diphenyl-1-picrylhydrazyl (DPPH) radical scavenging activity increased by about 15%] and antibacterial (the bacteriostatic zone for *E. coli* and *S. aureus* was 12–20 mm).

Various extracts or processing residues of animals and plants, such as cellulose, chitosan, propolis, protein, gallic acid, resveratrol, curcumin, and essential oils are often used in the blending modification of starch (Table 3) [108,141,155,156]. They have a wide range of sources and low cost, which could enhance the stability, mechanical, and barrier properties of starch-based materials, and even give them antioxidant, antibacterial, or ultraviolet light-shielding performances. For instance, Ali et al. [141] increased the mechanical properties (e.g., Young’s modulus, tensile strength, stiffness, and drop impact strength) of hydroxypropyl high-amylose starch-based films by adding pomegranate peel ground powder, and endowed the films with an inhibitory effect on the growth of *S. aureus* and *Salmonella*.

Chitosan is often uniformly blended with small molecular additives (e.g., glycerol, essential oils, and other plant extracts), or with other natural polymers (e.g., other polysaccharides, proteins, and lipids) to improve the comprehensive properties of chitosan-based composites (Table 3) [51,144,157,158]. Siripatrawan et al. [144] improved the functional properties of chitosan-based edible films by incorporating propolis containing high polyphenols, specifically enhancing the tensile strength, elongation at break, total phenol content, and antioxidant and antibacterial activities of the composite films, while reducing their oxygen and WVP. Likewise, Rambabu et al. [157] added mango leaf extract (MLE) to chitosan to significantly improve the tensile strength and surface hydrophobicity of the chitosan-based composite film and reduce its WVP, water solubility, and elongation at break. Moreover, the antioxidant activity of the composite film was higher than both the original chitosan and commercial PA/PE films (in which the antioxidant activity of the edible composite film containing 5% extracts was 56% higher than the PA/PE film).

In addition, polysaccharide-gum based composites with improved performance can be obtained by uniformly blending cellulose [113,145,159], starch [147,160,161], chitosan [146,148,162], another polysaccharide gum [149,163], as well as proteins [150,151], lipids [164,165,166,167], essential oils, and probiotics [64,152,153,168,169] with the original polysaccharide-gum matrix (Table 3). By adding nanocellulose (usually ≤ 5% *w*/*w*) to the agar matrix, Oun [145] and Shankar [159] et al. significantly improved the tensile strength, water vapor barrier and thermal stability of the agar-based edible films. Likewise, the addition of starch to agar by Phan [160] and Fekete [161] also enhanced the water resistance and water vapor barrier properties of the composite films and reduced the overall cost of the composites. When the amount of cassava starch was 20% (*w*/*w*), the WVP at 57–22% relative humidity differential of the composite film was reduced to about 3.33 × 10^−11^ g·m^−1^·s^−1^·Pa^−1^, which is 53.8% less than pure agar film. When the added amount was 50% (*w*/*w*), the WVP was about 2.99 × 10^−11^ g·m^−1^·s^−1^·Pa^−1^, which was 58.5% less than pure agar film [160]. Furthermore, the blending of chitosan (an alkali-soluble polysaccharide) and acidic polysaccharide gums produces electrostatic interactions, which makes the structure of the composite compact and without phase separation, thus, leading to better mechanical and barrier properties than a single material, and even improves the antibacterial and ultraviolet light-shielding properties of the composite [146,148,162]. Sodium caseinate was introduced into low methoxyl pectin by Eghbal et al. [150,151] to adjust the water content and absorption, as well as the mechanical and optical properties of the composites. The results indicated that the protein content affected the properties of the composites; the highest amount of complex coacervates of blending liquids was formed at a sodium caseinate/low methoxy pectin ratio of 2, at which the ζ-potential value was zero and the turbidity reached the highest value. While the ratio was 0.05, the Young’s modulus (182.97 ± 6.48 MPa) and tensile strength (15.64 ± 1.74 MPa) of the composite films were the highest, which were all higher than those of the pure pectin film. In addition, lipids (e.g., beeswax, shortening, and shellac) are the most effective natural substances to enhance the water and moisture resistances of polysaccharide gums [165,166,167]. Active extracts (e.g., various plant essential oils) could not only strengthen the thermal stability, and mechanical and barrier properties of polysaccharide gum-based materials, but also improve their antioxidative, antibacterial, and other functional characteristics [64,152,153,168,169].

## 4. Applications of Various Polysaccharide-Based Materials in Edible Packaging

Original polysaccharides can form self-assembled films, coatings, or microcapsules under the action of hydrogen bonding, van der Waals, or electrostatic forces, form hydrogels with a three-dimensional network structure through gelation, and form composites by combining them with other edible materials (e.g., proteins, lipids, probiotics, and other natural active small molecule substances), which can apply to different food packaging (Figure 1). The predominant use of polysaccharide-based edible materials is to serve as an auxiliary means of packaging. They can effectively delay the migration of water, gas, oil, and solute by providing a selective barrier, retain volatile flavor compounds and mechanical integrities of foods, improve treatment properties of foods, or even be used as non-toxic carriers of food additives (e.g., antioxidants, anti-browning, and antimicrobial agents) integrated into the packaging to improve the sensory properties of foods and extend their shelf lives.

**Figure 3:**
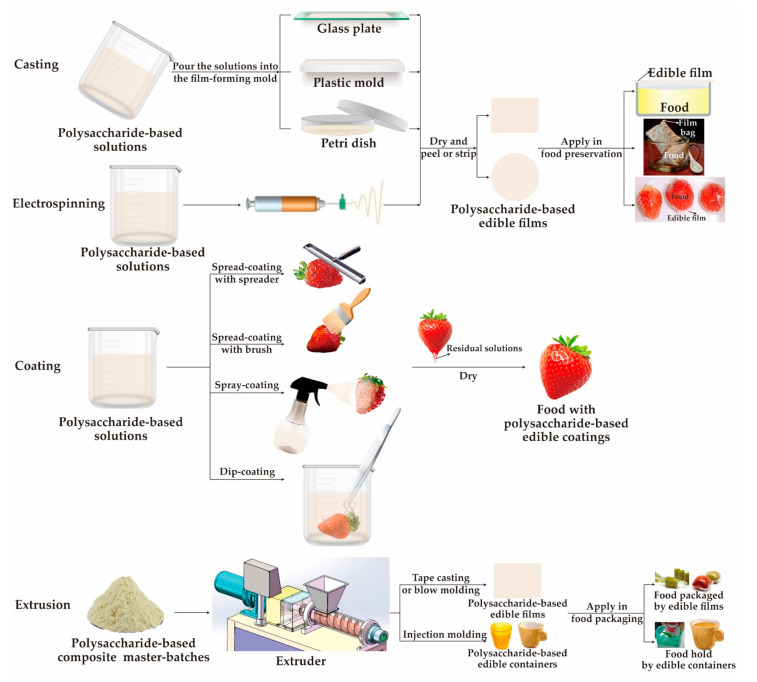
Different manufacture methods of polysaccharide-based edible packaging.

In the following section, the potential application of five polysaccharides (cellulose, hemicellulose, starch, chitosan, and polysaccharide gums) in targeting the edible packaging sector are briefly described. Whereas, Table 4 includes the main preparation methods, packaging forms, packaged objects, and packaging effects of the different polysaccharide-based materials; the main preparation methods are shown in Figure 3.

### 4.1. Applications of Cellulose

Cellulose is commonly applied in food packaging (e.g., fruits, vegetables, and oils) as edible films, coatings, and emulsions to protect the sensory qualities of foods and extend their shelf lives (Table 4). Rhimi et al. [98] added cypress seed extract to an HPMC matrix and prepared edible composite films using the casting method, and then applied them in olive oil packaging (as shown in Figure 3). The results indicated that compared with pure HPMC films, the tensile strength of the composite films was significantly improved (up to 15.13%) and the WVP was reduced (24.66% at most), which slowed down the oxidation of olive oil during 23 days storage. The lowest WVP, greatest opacity, and highest antioxidant capacity of the composite films were obtained with the highest extract concentration. Therefore, the peroxide value of olive oil sealed with composite films (containing 2% *w*/*v* extract) after accelerated storage for 11 days was 10 times lower than when sealed with pure HPMC films.

**Figure 4:**
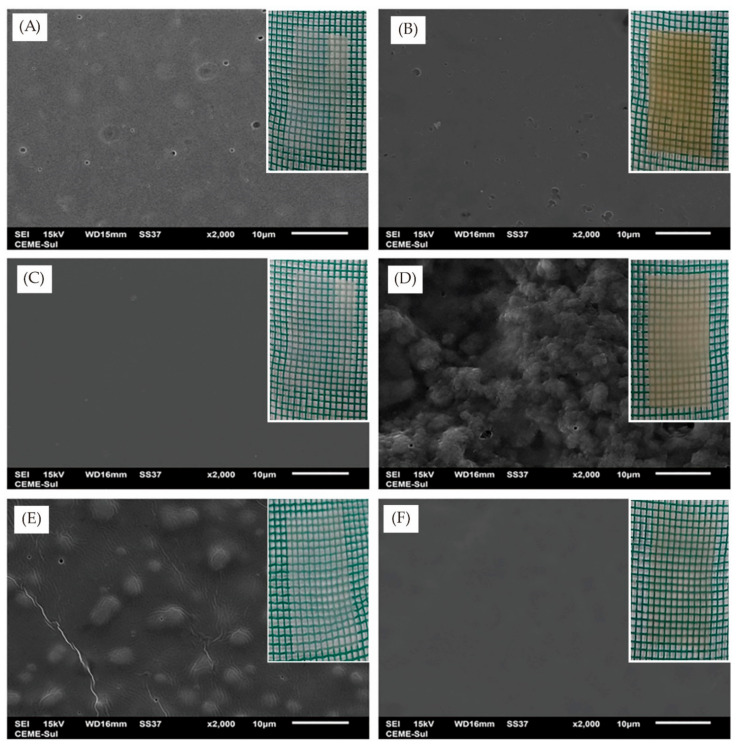
Scanning electron microscopy images (×2000) and physical photos of different edible films. (**A**) Collagen film; (**B**) Whey protein film; (**C**) Methylcellulose film; (**D**) Collagen/whey protein blend film; (**E**) Collagen/methylcellulose blend film; (**F**) Whey protein/methylcellulose blend film. (Adapted with permission from Filipini [91]; published by John Wiley and Sons, 2020).

**Figure 5:**
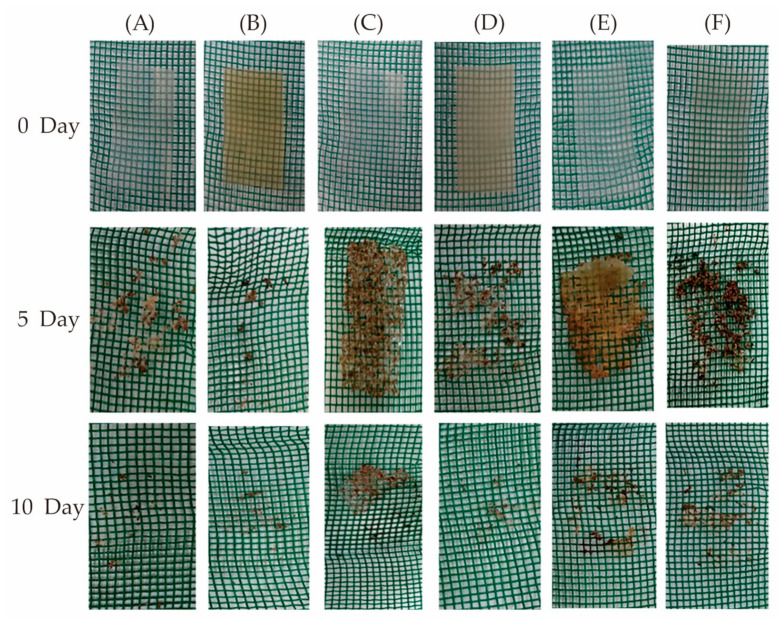
Biodegradability in the soil of different edible films. (**A**) Collagen film; (**B**) Whey protein film; (**C**) Methylcellulose film; (**D**) Collagen/whey protein blend film; (**E**) Collagen/methylcellulose blend film; (**F**) Whey protein/methylcellulose blend film. (Adapted with permission from Filipini [91]; published by John Wiley and Sons, 2020).

**Figure 6:**
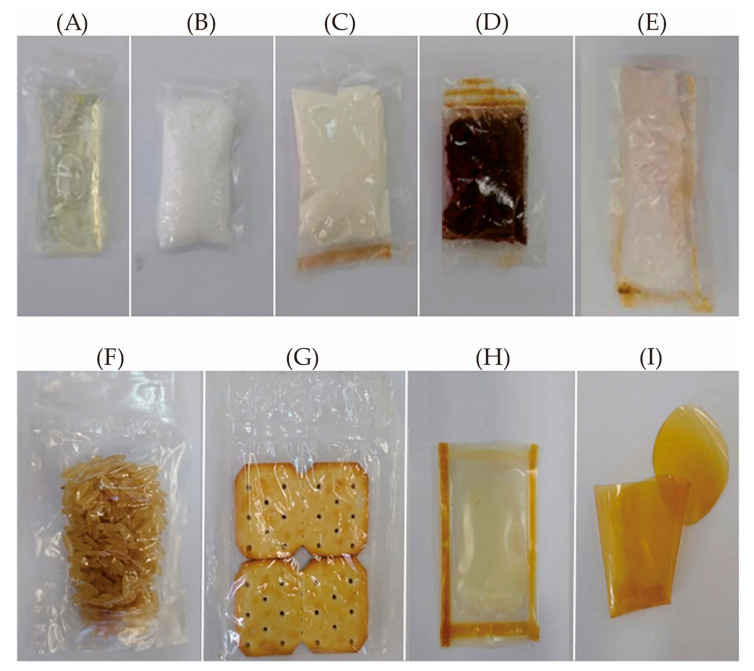
Prototype photos of different edible packaging. From (**A**–**G**) are methylcellulose sachets containing soybean oil, salt, whey protein, powdered coffee, powdered juice, rice, and cookies, respectively; (**H**) Whey protein/methylcellulose edible sachet containing oil; (**I**) Whey protein edible film for the coffee capsule. (Reproduced with permission from Filipini [91]; published by John Wiley and Sons, 2020).

It is also noteworthy that cellulose is usually added to other edible materials as a reinforcing or toughening agent to improve the properties of composites. In the blends with collagen and whey protein, methylcellulose was responsible for the increase in tensile strength, water vapor barrier, and thermal properties. While, the prepared methylcellulose-based edible materials (Figure 4) could maintain their integrity for months, be completely biodegraded in 10 days in soil (Figure 5), and when immersed in hot or cold water showed total solubilization in around 30 s upon manual shaking [91]. The edible packaging has immense potential applications in soluble sachets for powdered foods, as well as oil containers and capsules for instant foods (Figure 6). Furthermore, the addition of cellulose nanocrystals to soybean protein could improve the tensile strength and barrier properties (the static water contact angle increased, and the moisture content, WVP, and reduced oxygen permeability) of the edible composite film, and enable the film to obtain ultraviolet light-shielding performance on the premise of appropriate transparency [184]. In addition, the creaming stability and ability to form an elastic gel-like network of beeswax-in-water (O/W) Pickering emulsions could be improved by blending with cellulose nanofibrils/carboxymethyl chitosan. Meanwhile, the complex edible films cast by modified emulsions had good tensile strength (5.0 MPa at a strain of 2.2%) and low WVP (<2 × 10^−7^ g∙h^−1^∙m^−1^∙Pa^−1^), and could inhibit the growth of *S. aureus* and *E. coli*, a promising application for antiseptic and fresh-keeping packaging for berry fruits [185].

### 4.2. Applications of Hemicellulose

Hemicellulose is usually used in edible packaging as films, coatings, or modifying additives, which is like cellulose (Table 4). Taking KGM as an example, Yan et al. [170] introduced pullulan into the KGM matrix to cast edible composite films for strawberry preservation. They showed that the mechanical and barrier properties of the composite films were markedly enhanced because of the intermolecular interaction between KGM and pullulan; 1% (*w*/*v*) KGM/pullulan (with a mass ratio of 2:1) composite film significantly decreased the weight loss and maintained the titratable acidity, soluble solids, ascorbic acid, and skin color on strawberry preservation, thus slowing fruit aging, improving the quality during storage, and extending their shelf life to 14 days. Hashemi et al. [174] blended saffron petal extract with a KGM matrix to cast edible complex films, while coating fresh-cut cucumbers (as shown in Figure 3). The results indicated that saffron petal extracts markedly improved the transparency and moisture content of the complex films, reduced their WVP, and even endowed them with promising antioxidant and antimicrobial properties. Furthermore, this composite coating reduced mesophilic bacterial and fungal populations during cucumber storage (in which 4% extracts were considered as the most effective additives), improved the soluble solids content, antioxidant activity, and soluble phenols of coated sliced cucumbers, thus decreasing their spoilage, maintaining their quality features, and prolonging their shelf lives. Wang et al. [18] introduced zein into KGM matrix by electrospinning to form stable homogeneous nanofibril films, which the hydrophobicity was improved (SWCA of the composite film increased from 7.5° to 57.5°). Furthermore, they added curcumin into the above nanofibers to form a functional nanofilm with advanced antioxidant (scavenging activity increased about 15%) and antibacterial (a large inhibitory zone of 12–20 mm for *E. coli* and *S. aureus*) activities, as well as better thermal stability, water resistance and tensile strength.

### 4.3. Applications of Starch

Starch is often compounded with other edible materials to fabricate edible films or coatings, which are widely used in different food packagings, such as fruits, vegetables, meat, seafood, confectioneries, cakes, and pastries to block the migration of oxygen and grease and help improve the appearance, texture, and processing performance of foods (Table 4). Go et al. [175] added rosehip extracts to rye starch matrix to cast edible composite films and applied them in chicken breast packaging. The flexibility, optical properties, and antioxidant activity of the composite films were improved, and the highest ABTS and DPPH radical scavenging activities were observed in films containing 1.0% extracts (96.87% and 80.22%, respectively). Moreover, chicken breasts packaged with these films had lower peroxide and thiobarbituric acid reactive substance values than those packaged with original rye starch film, as well as the non-packaged control, suggesting that the edible composite films could effectively inhibit lipid oxidation and prolong its shelf life. Likewise, incorporating maqui berry extract [181], carvacrol, and chitosan [186] in starch-based edible composites (e.g., edible films and coatings) retarded lipid oxidation in fish, ham, and other foods, inhibited the growth of foodborne pathogens, and extended the shelf life of foods. Qin et al. [176] added *Lycium ruthenicum* Murr anthocyanins to cassava starch to manufacture a freshness indicator film with both intelligent pH sensitivity and edibility for pork packaging. The results showed that the barrier ability, tensile strength, and antioxidant activity of the composite film were improved by hydrogen bond interactions between anthocyanins and starch chains. Moreover, this composite film achieved real-time and visual monitoring of pork freshness based on its color change with pork quality during storage.

Furthermore, a significant difference from other polysaccharides is that original starch exposed to shear and high temperature (supplemented with water and processing aids) could be converted into thermoplastic starch-based materials, and then various starch-based edible packaging containers (e.g., film, cup, tray, and plate) can be obtained through extrusion, compression, or injection molding (Figure 3) [17,140,187,188].

### 4.4. Applications of Chitosan

Currently, chitosan-based edible packaging (as a film and coating) has been widely used in the packaging of fruits (e.g., strawberries, apples, kiwi, and grapes), vegetables (e.g., tomato, pepper, and eggplant), meats, and nuts to retain food quality and prolong their shelf life (Table 4) [56,171,177,189,190,191,192]. These edible packages mainly achieve food preservation by reducing the transpiration rate, delaying browning or lipid oxidation, and inhibiting the growth of spoilage microorganisms.

Divya et al. [56] coated chitosan nanoparticle solutions on the surfaces of tomatoes, chilies, and brinjals using the dip-coating method (Figure 3). The edible coatings had a good inhibitory effect on *Rhizoctonia solani*, *Fusarium oxysporum*, *Collectotrichum acutatum*, and *Phytophthora infestans* during 5 days of storage, had significant antioxidant activity, reduced the weight loss of these vegetables, and prolonged their shelf lives. Perdones et al. [189] applied chitosan-lemon essential oil dip-coatings to strawberry preservation. The results indicated that these edible coatings could control strawberry fungal decay during storage and affect the metabolic pathways and volatile profile by promoting the formation of esters and dimethyl furfural and incorporating terpenes into the fruit volatiles in a short time. Likewise, Dini et al. [177] packaged beef loins in chitosan-based edible films containing cumin essential oil nanoemulsions supplemented with irradiation treatment. The results showed that the edible composite films could withstand low-dose gamma irradiation at 2.5 kGy, while inhibiting the growth of *L. monocytogenes*, *E. coli* O157:H7, and *Salmonella typhimurium* in beef loins during the 21/days refrigerated storage, and slowed down the increasing level of total volatile basic nitrogen and pH value of beef, thus effectively enhancing the microbiological safety, quality, and storage life.

### 4.5. Applications of Polysaccharide Gums

Polysaccharide gums (e.g., pectin, alginate, carrageenan, and agar) are commonly used in edible packaging as gels, films, and coatings for food preservation of fruits (e.g., apple, peach, cherry), vegetables (e.g., tomato, papaya, and lettuce), meats, and seafood (Table 4), and even have commoditized packaging of pure water and other beverages. These polysaccharide-gum based edible materials could effectively reduce the dryness degree of food surfaces, prevent food from water loss and atrophy, and are beneficial to slow down lipid oxidation and surface discoloration of foods, as well as inhibit the reproduction of spoilage microorganisms, thereby extending the shelf life of foods [19,70].

López et al. [169] added green tea extract to an agar solution containing glycerin and glucose to prepare the substrates by casting, and then coated the substrates with probiotic strains (*Lactobacillus paracasei* L26 and *Bifidobacterium lactis* B94) to acquire the edible composite films and further apply in hake packaging. The results showed that during 15 days of storage, the edible composite films effectively inhibited the growth of spoilage microorganisms, especially the H_2_S-producing bacteria, causing a decrease in TVB-N, trimethylamine nitrogen (TMA-N), and pH value of the hake, and increased the beneficial lactic acid bacteria, thus leading to its shelf life extension for at least a week. Additionally, maltodextrin/calcium alginate edible casting films containing Tinospora cordifolia extracts were fabricated by Kalem et al. [179] and then used as casings substitutes for goat meat sausages. It was found that edible films with antibacterial and antioxidant properties could significantly reduce the production of thiobarbituric acid reacting substances and free fatty acids in sausages during storage, inhibit the reproduction of microorganisms (total plate, psychrophilic, and yeast and mold), and maintain the sensory quality of goat meat sausages. Similarly, the cooked ham portions were dipped in iota-carrageenan-based coating solutions containing rosemary extract, ascorbic acid, calcium chloride, α-tocopherol, and glycerol by Carocho et al. [178] for food preservation. The results showed the edible coating based on the above solutions inhibited the growth of microorganisms and retained the sensory quality of hams over the 15-days of storage.

## 5. Safety Risk Assessment of Polysaccharide-Based Edible Packaging

Edible packaging serves to protect food and act as a ready-to-eat “food”, which provides valuable nutrients and energy [193]. In theory, food-grade polysaccharides made from natural edible constituents used in most studies are non-toxic, and edible packaging prepared from these polysaccharides could be consumed by animals or humans without health risk [15]. However, to be edible actually, the materials (including substrates and additives) used in the formulations should be green, non-toxic, safe and meet applicable regulations or standards (e.g., GRAS—Generally Recognized as Safe by the FDA-U.S. Food and Drug Administration).

Uncertainties and knowledge gaps on the possible health effects and long-term safety of polysaccharides and their modifying additives, when used in edible packaging, are still the most important concern. To date, very few studies have been published regarding the effects of polysaccharides-based edible packaging upon ingestion, and the absorption, distribution, metabolism, and excretion after oral exposure, and the potential interactions of polysaccharides with packaged food components [194]. Most edible films and coatings, discussed in this review, focus on the preparation and characterization of materials, with little follow-up food safety risk assessment.

Therefore, polysaccharides-based edible packaging must be exhaustively studied, they are easier to transfer constituents into foods than petroleum-based polymers. The first step in assessing the potential hazard of polysaccharides-based packaging for a comprehensive risk assessment, in terms of consumer safety, is to evaluate their potential migration into food (usually according to Regulation (EU) No. 10/2011 on plastic materials and articles) [195]. In particular, the solubility of polysaccharides that migrate in the food matrix and/or upon gastrointestinal passage is a crucial factor.

In addition, toxicological risk and dietary exposure assessment are important for polysaccharides edible packaging. Barreto et al. [196] prepared two kinds of onion (*Allium cepa* L.) puree-based edible films by casting, namely unwashed hydrothermally treated pulp (HTP) and washed hydrothermally treated pulp (W-HTP), and then assessed their genotoxicological safety. The cellular viability demonstrated that HTP films showed greater cytotoxicity than W-HTP films; and the mutagenic activity indicated that both HTP and W-HTP films were not able to statistically increase the frequencies of the biomarkers for chromosome damage (micronucleus test) at the tested concentrations. However, the HTP films showed signs of mutagenicity in the Ames test (gene mutations), suggesting caution in their use. Therefore, W-HTP onion-based edible films are harmless and possess safety potential application in food packaging, supporting the first level of evidence. For the additives, Sohrabi et al. [197] evaluated the potential cyto-genotoxicity of ascorbyl palmitate (AP, a widely used food additive) on Human Umbilical Vein Endothelial Cells (HUVECs). The results indicated that the growth of HUVECs was decreased upon treatment with AP in dose-and time-dependent manner, and AP induced apoptosis by up-regulation of caspase-3, 9 and down-regulation of Bcl-2 ratio. Therefore, AP application in the edible packaging industry should be carefully considered.

**Figure 7:**
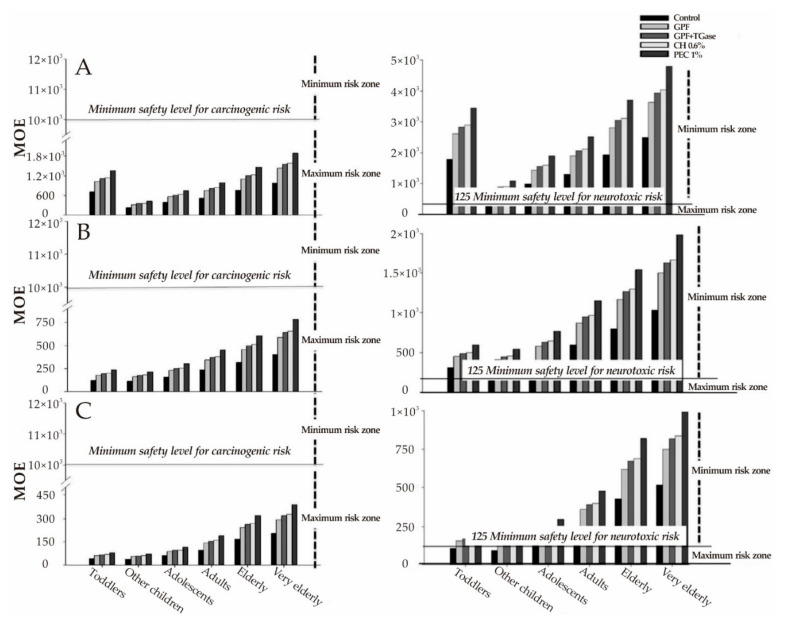
MOE values for carcinogenicity (**left panel**) and neurotoxic (**right panel**) of acrylamide through the consumption of French fries that were both uncoated and coated with hydrocolloid coating solutions. Samples were coated with different polysaccharides-based coatings made of PEC, pectin; and CH, chitosan. “Uncoated” represents the control sample dipped in distilled water, across different consumer age groups: (**A**) minimum, (**B**) median, and (**C**) maximum of consumption levels estimated from the 2015 EFSA report. (Adapted with permission from Al-Asmar [199]; published by MDPI, 2018).

Zheng et al. [198] prepared hydroxypropylated-Phosphated-modified glutinous rice starch and evaluated its safety through acute and 28-day repeated oral toxicity tests. The results showed that the modified starch possessed more than 10,000 mg/kg LD_50_ value, was belong to non-toxic. Moreover, its acceptable daily intake for a normal person (70 kg) should be less than 38,900 mg, which means that the recommended intake (RNI) is no more than 38,900 mg/d. Asmar et al. [199] dipped the potato sticks into chitosan or pectin hydrocolloid coating solutions before frying to reduce the acrylamide and oil content of French fries. Then, the Daily Intake (DI) (Table 5) and Margin of Exposure (MOE) (Figure 7) were further calculated by considering the six following age groups (as stated from EFSA) to estimate variations in risk assessment by applying coating solutions. The results showed that, compared with the control sample (reached highest acrylamide concentration 2089 µg·kg^−1^), the edible polysaccharides coating reduced the acrylamide content by 48% for pectin and >38% for chitosan, respectively. Moreover, the increasing MOE value indicated that recurring coatings could provide advantages to consumers, especially for the ones from 1 to 65 years old, and the pectin coating was the most effective.

Overall, a series of safety studies can be conducted on edible materials based on relevant regulations and standards (e.g., FDA for Preparation of Food Contact Notifications for Food Contact Substances-Toxicology Recommendations), such as composition analysis (including nutritional composition and possible natural toxic substances), hygienic tests (heavy metals, pesticide residues), and toxicological tests [including acute oral toxicity test, three genetic toxicity tests (Ames test, mammalian red blood cell micronucleus test and mouse spermatocyte chromosome aberration test), 90 d oral toxicity test and teratogenicity test], and further combined with the population, history of consumption, and the survey results of adverse reactions to assess the safety of polysaccharides-based edible packaging comprehensively.

## 6. Conclusions and Prospects

Edible packaging is a vital component of sustainable packaging. It significantly expands the source of packaging materials, reduces the dependence on non-renewable petroleum resources, and efficiently uses food processing waste. Polysaccharides are the major study objects of edible packaging materials. Considering the advantages and limitations of polysaccharides, researchers currently use various modifications to optimize the material’s comprehensive properties, such as film-forming, mechanical and barrier properties, and antioxidant and antibacterial activities. They have successfully developed a variety of polysaccharide-based edible packaging materials such as ink, microcapsules, coatings, films, and sheets, which are applied to food packaging. These materials can provide selective barriers to prevent the migration of water, gas, and lipid in the food-packaging system, effectively retain the flavor and nutrition of food, and extend its shelf life (e.g., fruits, vegetables, meat, aquatic products, nuts, confectioneries, and delicatessens, etc.).

In general, polysaccharide-based edible packaging plays a key role in the environmental protection of food packaging and the high value of food processing waste, and it is one of the best alternative non-renewable resources. Although numerous studies on polysaccharide-based edible packaging have been reported in the past 10 years, they are still mainly on the laboratory scale and are less industrialized. Herein, trends of research and application of polysaccharide-based edible packaging will mainly focus on the following four aspects:

(1) Development of more edible polysaccharide-based materials: To date, the primary sources of polysaccharide-based edible packaging are plants and animals. Microorganisms, such as bacteria and fungi are also a great potential source, especially for the development of marine microorganisms.
(2) Multi-functional modification of polysaccharide-based materials: Modification of materials based on practical application requirements is a hot topic in material science. Defects in mechanical properties and water resistance are difficulties in the application of polysaccharide-based materials. Whereas, hydrophilicity is a key factor influencing these performances. Therefore, selecting the most reasonable modification technology (e.g., high stability, low cost, safety, convenience, and easy industrialization) for new polysaccharide-based materials to improve their properties would be an important topic for future research on polysaccharide-based edible packaging. Therefore, the design of the binding mode between polymer chains, the design of monomer molecular group structures, and the realization of more functional effects according to different food requirements (e.g., flavor regulation, acid and alkali resistance, amphiphobicity, and controlled release of functional factors) are the development trend of the future modification of polysaccharide-based materials.
(3) Application expansion and comprehensive evaluation of polysaccharide-based edible packaging: To conduct engineering research on cost reduction and large-scale production, and to evaluate the economic, environmental, and social benefits of polysaccharide-based edible packaging (the life cycle sustainability assessment theoretical model is recommended here), is the only way for the application and promotion of polysaccharides in packaging and food fields in the future.
(4) A deeper knowledge and practice of safety risk assessment for polysaccharides: Understand the potential exposure of polysaccharides through migration into food, the interaction of polysaccharides with food constituent, and their effects upon ingestion, which could verify polysaccharides-based edible packaging safety for commercial purposes and provide a reference for dietary reference intake of residents.

## Figures and Tables

**Table 1:** Sources, compositions, structures, and outstanding characteristics of five polysaccharides for edible packaging application.

Polysaccharides		Sources	Molecular Structure Characteristics	Functional Advantages
Cellulose		Major: wood and cotton Minor: certain peels, husks, bagasse, algae, vegetables, tunicates fungi, invertebrates, and bacteria [16,24,25]	Comprise anhydroglucose units connected by β-glycosidic bonds Contains numerous hydroxyl groups [26]	Good chemical stability, gelation, and film-forming properties Good mechanical properties, and barrier capacities to oxygen and lipids Renewable, biodegradable, biocompatibility Soluble dietary fiber and food additive [13,16,27] □ Compared with ordinary cellulose, nanocellulose has a higher elastic modulus, tensile strength, crystallinity, lower coefficient of thermal expansion, large specific surface area, high reactivity, and small size effects [28]
Hemicellulose	Xylan	Major: hardwoods, gramineous plants Minor: certain crops and their processing by-products [29,30,31,32]	Composed of (1→4) bonds connected to the main chain of β-D-pyranose units and different side groups connected by (1→2) and/or (1→3) bonds Contains numerous hydroxyl groups [29,30,32]	Good gelation, and film-forming properties Good mechanical and gas barrier properties (But these properties are slightly worse than those of cellulose) Renewable, biodegradable, biocompatibility Soluble dietary fiber and food additive [16,29,30,32]
	Glucomannan	Softwoods, tubers and seeds of *Amorphophallus konjac* plants [30,31,33]	Composed of D-glucopyranosyl and D-mannopyranosyl connected by β-(1→4) bonds Contains numerous hydroxyl groups [16,30,31]	
Starch	Amylose	Major: corn, rice, wheat, cassava, and potatoes [34,35] Minor: banana, mango, breadfruit [34,35], oca [36], jackfruit and lotus seeds [37], and pineapple stems [38]	Composed of α-D glucose connected by α-(1→4) glycosidic bonds; has no branched structure or only a small amount of branched structures connected by α-(1→6) glycosidic bonds Only hydrophobic hydrogen atoms inside the helix structure, and hydrophilic hydroxyl groups outside it [16,39,40,41]	Good mechanical properties, oxygen barrier property, and processability Renewable, biodegradable, recyclable, biocompatibility Low processing cost Food additive [15,42,43] □ Gelatinize, regenerate, swell, and a certain proportion of starch aqueous solution behaves as non-Newtonian fluid (The above characteristics are not available in polysaccharides such as cellulose, hemicellulose, chitosan and alginate); and semi-permeable to carbon dioxide [41,44,45,46] □ Worse gelation, film-forming properties, and transparency if compared to cellulose, hemicellulose, chitosan, and polysaccharide gums [15,16]
	Amylopectin		The main chain is composed of α-D-(1→4) glycosidic bonds, and the side chain is composed of α-(1→6) glycosidic bonds; the structure is more complex and arranged radially in a concentric form Contains numerous hydroxyl groups [44,47,48]	
Chitosan		Major: the shells of crustaceans such as shrimps, crabs, insects Minor: the cell walls of lower plants, bacteria, and fungi [49]	Composed of N-acetyl-D-glucosamine and D-glucosamine (occupies a larger proportion, generally > 55%) connected by β-(1→4) glycosidic bonds Contains numerous amino and hydroxyl groups, and a few acetylamino [16,50,51]	Good gelation, film-forming properties and processing suitability Good mechanical, oxygen and lipids barrier, and adsorptive properties (The tensile strength and swelling power of chitosan films prepared at higher drying temperatures and solute concentrations improved relatively) Renewable, biodegradable, biocompatibility Food additive [51,52,53,54] □ The high specific surface area, large aspect ratio, and small size effect of nano-chitosan can further improve the biological activity, biocompatibility, and adsorption properties of ordinary chitosan [55,56] □ Good antioxidant activity; and excellent antimicrobial activity, with effective inhibition of most gram-negative and -positive bacteria and fungi (These properties differ from those of cellulose, hemicellulose and starch) [57,58]
Polysaccharide gums	Pectin	Major: fruit and vegetable processing residues such as citrus peel, apple peel, sweet potato residue, and beet residue [59] Minor: the peels of passion fruit [60], lime [61], dragon fruit [62], fig [63], grapefruit [64], pomegranate [39], lemon [65], and hawthorn [66]; and sunflower heads without seeds [67], *Premna microphylla* Turcz leaves, and Creeping fig seeds [68]	An acidic heteropolysaccharide composed of D-galacturonic acid and other neutral sugars; the fine structure of the domain has not yet been fully clarified [15] Contains numerous hydroxyl and carboxyl groups [63,66] Complex metal ions such as Fe^2+^ or Cu^2+^; enhance the activity of antioxidant enzymes such as superoxide dismutase and catalase [63,66]	Better gelling and film-forming properties than cellulose and starch Better water-retaining properties and transparency than cellulose and starch Good oxygen and lipids barrier properties (Their products have oil-, grease-, and odor-proofing capabilities and can effectively slow down the oxidation of food lipids) Renewable, biodegradable, biocompatibility Soluble dietary fiber and food additive (e.g., water sacrificial agent) [15,16,60,69,70,71] □ Pectin and its derivatives extracted from the peel of certain fruits (e.g., fig, lemon, apple, and hawthorn) have antioxidant and antimicrobial activities; furthermore, pectin has a weak antibacterial effect, but its degradation products (especially pectin enzymatic hydrolysis products) have an obvious inhibitory effect on common foodborne pathogens such as *Staphylococcus aureus*, *Escherichia coli*, and *Vibrio parahaemolyticus* [63,65,66,72,73] □ Alginate exhibit polyanion behavior in an aqueous solution and have a certain amount adhesion [16,74] □ The commonly used agar for packaging is agarose, and its molecules can interact through hydrogen bonds to form a continuous and firm network structure [71]
	Alginate	Major: cell wall and intercellular mucilage of brown algae such as *Laminaria*, *Kelp*, *Durvillaea potatorum*, and *Sargassum* Minor: some *Pseudomonas*, nitrogen-fixing bacteria, and other bacteria that can produce mucous capsules [15,16,74,75,76]	A long-chain linear copolymer connected by β-D-mannuronic acid and α-guluronic acid, according to (1→4) bonds Contains numerous -COO- groups Its products usually include sodium alginate, potassium alginate, calcium alginate, zinc alginate, and magnesium alginate [16,74,76,77,78]	
	Carrageenan	Cell walls of marine red algae, such as *Eucheuma*, *Chondrus*, *Gigartina*, *Gelidium*, and *Hypnea* [15,79]	A linear galactosan composed of sulfated or non-sulfated galactose and 3,6-dehydrated galactose alternately connected by α-(1→3) and β-(1→4) glycosidic bonds Divided into seven types (e.g., κ, ι, λ, γ, ν, ξ, and μ-type) according to the different binding forms of sulfate esters Contains numerous hydroxyl groups [15,79]	
	Agar	Marine red algae, such as ferns, asparagus, laver, *Gelidium*, and *Gracilaria* [80]	A galactose polymer composed of agarose and agaropectin Agarose is a non-ionic polysaccharide without sulfate (salt) and comprises 3,6-dehydration-α-L-galactose and β-D-galactose residues alternately connected by (1→3) glycosidic bonds Contains numerous hydroxyl groups [15,69,80]	

**Table 2:** Chemical modification methods and effects of various polysaccharides.

Polysaccharides	Modification Methods	Edible Packaging Materials	Th	MC/%	WS/%	TS	EB/%	WVP	Functional Characteristics
Cellulose	Methylation (etherification)	Methylcellulose (MC) [89]	0.041	27.3	100	55	36	2.78 × 10^−10^	Better water solubility and mechanical properties than native cellulose
		MC [90]	0.048		98.9	31.4	16.2	7.95 × 10^−11^	
		MC [91]	0.062		100	15.78	15.4	1.19 × 10^−4^	
	Carboxymethylation	Carboxymethyl cellulose (CMC) [92]	0.142	16.55		10.48	42.37	1.198 × 10^−3^	Improve transparency, thermal stability, salt tolerance and acid resistant properties
		CMC [93]	0.097		21.19	0.23	60.21	7.41 × 10^−7^	
		CMC [94]	0.05			56	6.5	11.18 × 10^−11^	
		CMC [95]	0.070	22.71	75.08	14.18	10.54	3.36 × 10^−10^	
	Hydroxyethylation (etherification)	Hydroxyethyl cellulose (HEC) [96]	0.07		93.26			WVTR: 18.94	The water retention capacity of HEC is higher than that of MC, and it has a good thickening effect The dispersion of HEC is worse than that of MC and HPMC
	Hydroxypropylation (etherification)	Hydroxypropylated cellulose (HPC) [87]	0.04			7.0	7.5	7.52 × 10^−5^	Enhance mechanical and barrier properties
		Hydroxypropyl methyl cellulose (HPMC) [92]	0.110	24.54		19.25	37.56	95.66 × 10^−5^	
		HPMC [97]			100	33.0	13.4	1.34 × 10^−10^	
		Average methoxyl content/hydroxypropyl content (M/HP): 3.05 [83]	0.025			30.83	6.06	2.036 × 10^−4^	
		M/HP: 2.26 [83]	0.044			52.13	11.89	4.136 × 10^−4^	
		M/HP: 3.05 [83]	0.030			67.28	17.37	2.566 × 10^−4^	
		HPMC [98]	0.079			53.02	10.32	6.85 × 10^−5^	
	Acetic acid esterification	Acetylated cellulose (DS 0.54) [82]	0.04–0.12						Surface -OH are replaced by non-polar -COCH_3_ Better hydrophobic property (SWCA 73° > native cellulose 48°) and thermal stability
	Cross-linking	Tea catechins-cross-linked MC [90]	0.060		25.5	73.7	2.8	2.84 × 10^−11^	Light barrier, antioxidant and antibacterial properties
		Dialdehyde carboxymethyl cellulose (DCMC) crosslinkedfeather keratin (FK) [99]	0.09–0.15	17.9	43.8	2.1	26.8	3.3 × 10^−10^	Covalent bonds and hydrogen bonds occurred between FK and DCMC DCMC could slightly improve the water resistance, water vapor barrier property and flexibility, whereas reducing tensile strength
	Graft copolymerization	MC-g-2-hydroxyethyl methacrylate [100]	0.025					8.6 × 10^−5^	Puncture strength was 282 N/mm; Puncture deformation was ≈5.501 mm Grafted MC-based films’ surface appeared better smoothness
Hemicellulose	Carboxymethylation	Carboxymethyl xylan (DS 0.30) [101]	0.052			28.0	1.6	1.6 × 10^−5^	OP: 47 × 10^−9^
	Hydroxypropylation	Hydroxypropylated birch xylan [87]	0.04			39.0	4.5	1.5 × 10^−5^	Hydroxypropyl groups acted as inner plasticizers Better barrier and mechanical properties; OP: 6.5 × 10^−9^
	Esterification	2-dodecenyl succinic anhydride-modified xylan (DS 0.31) [101]	0.057			29.3	6.0	0.69 × 10^−5^	Better barrier and mechanical properties; OP: 42 × 10^−9^
	Acetylation	Acetylated bleached hemicellulose (DS 1.8) [82]	0.04–0.12			44.1	5.7		Better hydrophobic (SWCA 72° > Unmodified 57°), thermal stability and mechanical properties
	Cross-linking	Add citric acid into wheat straw hemicelluloses matrix containing cellulose nanocrystals [102]		7.0–7.5	47.41	9.76	3.94	4.09 × 10^−4^	Citric acid worked as crosslinker and plasticizer Enhanced modulus, elongation, water resistance, and water vapor barrier property
Starch	Carboxymethylation	Carboxymethyl starch (as functional master batch or raw material) [103]							Does not tend to retrogradation (recrystallization) Higher thermal stability and water solubility
	Hydroxypropylation	Hydroxypropylated rice starch (Molar substitution: 0.022–0.033) [104]			4.46–5.97	3.88–5.53	79.57–132.58	4.19–5.75 × 10^−5^	Improved swelling capacity, viscosity and paste clarity Higher elongation at break, water vapor permeability, film solubility, and transparency
	Acetylation	Acetylated cassava starch [96]	0.04		28.73			WVTR: 12.84	Reduce the gelatinization temperature Increase the hydrophobicity and tensile strength
	Esterification	Thermoplastic/succinated cassava starch (as functional master batch or raw material) [84]							The starch developed B- and V-type structures Smoother, continuous, and homogeneous starch matrix as the percentage of 2-Octen-1-ylsuccinic anhydride (OSA) increased, including numerous partially gelatinized granules The incorporation of OS groups via reactive extrusion imparts better thermal stability (improved by 20%)
		Starch-laurate esters (DS 0.45–2.92) [105,106]							Lauric acid (C_12_) replaces -OH groups, and the modified starch shows melting thermoplastic behavior and hydrophobicity Better thermal stability, clarity, mechanical properties (the elastic storage modulus could reach 226 MPa at room temperature) With DS increasing, glass transition temperature and tensile strength increase while melting temperature decreases
	Cross-linking	Add citric acid (as crosslinker and plasticizer) into carboxymethyl potato starch (DS 0.5) matrix [107]	0.2–0.3		58	0.16	26		Best mechanical performance and thermal stability containing 30% (*w*/*w*) citric acid; E: 0.65 MPa; Glass-transition temperature: 58 °C An excess of citric acid could lead to carboxymethyl starch hydrolysis
		Add citric acid into corn starch (DS ≈ 0.98), and then blended with grape juice [108]	0.17		59	0.24	63.68	4.7 × 10^−4^	Citric acid acted as crosslinker and plasticizer Better transparency, water barrier property and elongation at break than native film OP: 6.2 × 10^−9^
		Add sodium trimetaphosphate into corn starch (DS 0.95), and then the modified starch was blended with grape juice [108]	0.17		55	0.38	16.47	3.84 × 10^−4^	OP: 2.51 × 10^−9^ Sodium trimetaphosphate acted as crosslinker Better barrier properties and tensile strength
	Click chemistry: Cu(I) catalyzed azide-alkyne [3 + 2] cycloaddition (CuAAC)	Amphiprotic starch derivatives linked 1,2,3-triazole (as antibacterial raw material) [109]							Enhanced antibacterial activities for *E. coli* and *S. aureus*, and inhibitory activity decreased in the order: CBTST > CMTST > BMTST > HMTST > starch; the inhibitory index of CBTST attained 97% above at 1.0 mg/mL 1,2,3-triazole substituted groups with stronger electron-withdrawing ability relatively possessed greater antibacterial activity
Chitosan (CS)	Carboxymethylation	Carboxymethyl chitosan (DS 0.49) [110]	0.159			21.25	42	6.65 × 10^−11^	Biodegradable and soluble over a wide range of pH High viscosity
	Graft copolymerization	Ascorbic acid was chemically grafted into CS backbones to form chitosan ascorbate (DS 0.88) [111]	0.067	10.7	43.0	22	12.0	6.3 × 10^−10^	Improved light barrier, water solubility, and water vapor barrier Antioxidant activity (EC_50_ < 0.025)
		Chitosan acetate (DS 0.60) [111]	0.070	24.3	20.4	43	31	8.6 × 10^−10^	Better thermal stability and mechanical properties; EC_50_ > 1.60
	Cross-linking	Add fulvic acid (as crosslinker) into konjac glucomannan/chitosan matrix [33]				57.79	21.04	5.25	Better thermal stability, optical, water vapor barrier properties, and tensile strength Improved antimicrobial activity (when the addition of fulvic acid ≤ 0.01% *w*/*w*)
Polysaccharide gums	Carboxymethylation	Carboxymethyl agar (CMA) [112]							Gel skeleton microstructure of CMA was porous network structure, and the pore size of CMA became smaller and denser with the increase of DS Hygroscopicity increased, but thermal stability decreased
	Cross-linking	Add calcium chloride (as crosslinker) into citrus pectin/CMC composite matrix [113]				468	10.6	4.45 × 10^−11^	Carboxyl group from pectin are mainly involved in interactions with CMC, whereas -OH groups are mainly involved in self-associated hydrogen bonding of biopolymers Better thermal stability and mechanical properties (E: 4.4 ± 0.66 GPa), but worse water vapor barrier

Note: DS: Degree of substitution; Th: Thickness, mm; MC: Moisture content; WS: Water solubility; TS: Tensile strength, MPa; EB: Elongation at break; WVP: Water vapor permeability, g·m^−1^·s^−1^·Pa^−1^; WVTR: Water vapor transmission rate, g·h^−1^·m^−2^; OP: Oxygen permeability, cm^3^·m^−1^·d^−1^·Pa^−1^; SWCA: Static water contact angle; EC_50_: Antioxidant value against the DPPH radical (namely, the mass concentration of antioxidants produced a 50% scavenging effect against active free radicals), mg/mL; E: Young’s modulus; HMTST: 6-hydroxymethyltriazole-6-deoxy starch; BMTST: 6-bromomethyltriazole-6-deoxy starch; CMTST: 6-chloromethyltriazole-6-deoxy starch; CBTST: 6-carboxyltriazole-6-deoxy starch.

**Table 3:** Physical modification methods and effects of various polysaccharides.

Polysaccharides	Modification Methods	Th	MC/%	WS/%	TS	EB/%	WVP	Functional Characteristics
Cellulose	Blend CMC with gelatin and add *Dianthus barbatus* essential Oil [93]	0.100		9.86	0.16	68.37	2.19 × 10^−7^	More flexible Better antioxidant and antimicrobial activities
	Add dipalmitoyl lecithin liposomes loaded with quercetin and rutin to CMC matrix [133]	0.035–0.045						Antioxidant activity Sustained-release function (preserve poly- phenols and control their release)
	Add α-tocopherol and a mixture of polysorbate 80 and lecithin to CMC matrix [94]				44	18.5	12.45 × 10^−11^	More flexible Antioxidant activity and sustained-release function
	Add spent coffee grounds polysaccharides to CMC matrix [95]	0.070	21.63	50.52	26.04	6.84	3.36 × 10^−10^	Light barrier, antioxidant and antimicrobial properties
	Add cypress (*Cupressus sempervirens*) cone seeds extracts to HPMC matrix [98]	0.084			61.04	7.67	5.16 × 10^−5^	Light barrier and antioxidant properties
Hemicellulose	Add cellulose nanocrystals into wheat straw hemicelluloses matrix [102]		7.0–7.5	93.75	11.25	3.13	8.376 × 10^−4^	Improved tensile strength, modulus, water resistance, and water vapor barrier property
	Blend acetylated hemicellulose (DS 1.7) with acetylated nanocellulose (DS 2.34) [134]	0.250		17.67	10.59	15.49		Increasing DS and loading of acetylated nanocellulose, increased hydrophobicity (SWCA 68.29°) of composite and reduced its solubility in food simulants
	Blend konjac glucomannan (KGM) with microcrystalline cellulose [135]	40.53			5.12		WVTR: 3.38	Improved thermal stability, barrier and mechanical properties compared pure KGM film
	Add polydopamine functionalized microcrystalline cellulose into KGM matrix [135]	43.01			8.51		WVTR: 1.67	Better thermal stability, barrier and mechanical properties
	Add CS/gallic acid nanoparticles into KGM matrix [127]				42.50	26.61	11.25 × 10^−11^	Better thermal stability, water vapor barrier and tensile strength Obtain UV barrier and antimicrobial activity (*S. aureus* and *E. coli* O157:H7)
	Blend KGM with zein and add curcumin [136]				7.34			Better hydrophobic (SWCA: 32.6–57.5°), thermal stability and mechanical properties Good antioxidant (DPPH value: 42.6–51.48%) and antimicrobial activities
	Blend KGM with pectin [137]	0.048	17.91		15.75	22	1.76 × 10^−10^	Improved mechanical properties compared pure KGM or pectin film SWCA: 69.50°; DPPH value: 10.50%
	Add tea polyphenol into KGM/pectin matrix [137]	0.061	16.13		21.03	16.94	1.37 × 10^−10^	Better thermal stability, hydrophobicity, water vapor barrier, and tensile strength Improved antioxidant and antimicrobial activities (e.g., *E. coli* and *S. aureus*) SWCA: 88.43°; DPPH value: 50.46%
	Blend KGM with shellac [138]	0.106			13.8	20.5	11.28 × 10^−5^	Improved thermal stability, water resistance (SWCA 63.3°) and mechanical properties
Starch	Blend acetylated cassava starch with hydroxyethyl cellulose [96]	0.06		61.24			WVTR: 16.27	Films with higher concentrations of hydroxyethyl cellulose were thicker, more transparent and hygroscopic -OH groups in hydroxyethyl cellulose might have strongly bonded to the -COOH from acetylated starch, increasing the WS
	Blend carboxymethyl potato starch (DS 0.8) with carboxymethyl cellulose (DS 2.6) and add citric acid and glycerol [139]	0.2–0.3			3.4	29		E: 4.9 MPa Improve thermal, mechanical and hydrophilic properties
	Blend octenylsuccinated- (DS 0.0425) with native- sweet potato starch and add glycerol [120]	0.091	13.41	15.25	0.72	260	5.69 × 10^−11^	SWCA: 91.59°; Oil permeability: 0.149 ± 0.010 g·mm·d^−^^1^·m^−^^2^ Enhance moisture-proof property, elongation at break and transparency Damage tensile strength and surface morphology
	Blend acetylated- with native- corn starches and add glycerol to form thermoplastic corn starch [17]	0.129	9.26		23.99	6.14	1.20 × 10^−10^	Better barrier properties; OP: 2.57 × 10^−5^, CO_2_ Permeability: 3.32 × 10^−5^ cm^3^·d^−1^·m^−1^·Pa^−1^ Maintain mechanical properties
	Add CS into thermoplastic corn starch [140]	0.138			12.5	1.64	0.87 × 10^−9^	Higher UV absorption and opacity Better barrier and mechanical properties Antimicrobial property (e.g., *S*. *aureus* and *E*. *coli*)
	Add chitin into thermoplastic corn starch [140]	0.121			12.6	1.86	0.59 × 10^−9^	
	Blend rice starch with carboxymethyl chitosan (DS 0.49) [110]	0.143			18.5	35	4.70 × 10^−11^	Better transparency, thermal stability, and mechanical properties Delayed biodegradation
	Blend hydroxypropyl high-amylose starch with pomegranate peel [141]	0.11			24.32	9.39		Good antibacterial properties (*S. aureus* and *Salmonella*) Better mechanical properties (e.g., stiffness, modulus, tensile strength and drop impact strength); E: 611.79 ± 72.11 MPa, Energy at peak load: 3.69 ± 0.43 J
Chitosan	Blend CS ascorbate (DS 0.80) with MC [89]	0.044	21.9	61	35	24.4	2.93 × 10^−10^	Better water solubility, barrier and mechanical properties Maintain antioxidant activity (EC_50_: 1.30)
	Blend CS with carboxymethyl chitosan and add nisin [142]	0.048		45.4	9.2	19.8	7.65 × 10^−10^	Carboxymethyl chitosan possessed plasticizing effect, led to higher EB, lower TS, and thermal stability Nisin reduces transparency and mechanical properties, but improves antimicrobial activity for *Listeria monocytogenes* and water solubility Combination of CS with CMCS improves the antimicrobial activity
	Blend CS with carboxymethyl chitosan [142]	0.021		15.4	25.4	58.4	3.43 × 10^−10^	
	Add nisin into CS matrix [142]	0.043		37.5	11.4	15.3	6.35 × 10^−10^	
	Blend CS with gelatin and add thymol [143]	0.104					WVTR: 2.18	Antioxidant and antifungal properties
	Blend CS with starch and add thymol [143]	0.108					WVTR: 1.32	
	Blend CS with propolis extract [144]				17.5	12.1	0.578 × 10^−8^ OP: 0.21 × 10^−8^	Better gas barrier and mechanical properties Antimicrobial (e.g., *S. aureus*, *Salmonella Enteritidis*, *E. coli*, and *Pseudomonas aeruginosa*) and antioxidant activities
Polysaccharide gums	Blend agar with acid hydrolyzed cotton linter cellulose nanocrystals (which neutralized with NaOH) [145]	0.052			33.7	30.7	1.9 5 × 10^−9^	E: 0.72 ± 0.01 GPa; SWCA: 39.1° Better optical, thermal stability, mechanical, and water vapor barrier properties (When the addition of cellulose nanocrystals ≤5%)
	Blend agarose with CS [146]	0.013			42.35	16	6.95 × 10^−11^	Better hydrophobicity (SWCA: 97.7°) and mechanical properties, but slightly higher WVP
	Blend pectin (75–80% degree of esterification) with corn flour [147]	0.06	21.2	70.7	7.47		0.022 × 10^−7^	Improved mechanical, structural, thermal, and water vapor barrier properties Antioxidant activity, DPPH value: 13.97 ± 3.08%
	Blend CS (prepared from *Callinectes sapidus*) with (high methoxyl pectin (prepared from *Citruis sinensis Osbeck* peel) [148]	0.082	16.9		17.5	35	0.97 × 10^−15^	Better water vapor barrier and mechanical properties
	Blend gum tragacanth with locust bean gum [149]	0.047	13.07		20.28	1.10	0.83 × 10^−4^	Improved transparency, water barrier, and mechanical properties Decreased surface tension (53.97 ± 0.28 mN/m) could enhance the spreadability and coating integrity when applied to foods
	Blend low methoxyl with pectin sodium caseinate at pH 3 [150] and pH 7 [151]	0.040	14.5		15.64	9.35		Better E (182.97 ± 6.48 MPa) and TS Exist attractive interactions between the two negatively charged biopolymers Charge neutrality occurred for a sodium caseinate/low methoxyl pectin ratio corresponding to the maximal coacervation
	Add *Origanum vulgare* subsp. *viride* essential oil into basil seed gum [152]	0.060	17.92				3.69 × 10^−11^	Improved water vapor barrier Antioxidant and antimicrobial activities
	Add fish protein hydrolysate into agar matrix [153]	0.044		48.86	19.89	42.70	10.04 × 10^−11^	Higher mechanical properties, WVP, solubility, and yellowness Alcalase hydrolysate exhibited antimicrobial effect against five tested microorganisms (e.g., *Staphylococcus aureus*, *Yersinia enterocolitica*, *Aeromonas hydrophila*, *Debaryomyces hansenii* and *Listeria innocua*)
	Add clove essential oil into agar matrix [153]	0.061		20.86	10.16	3.93	9.37 × 10^−11^	Better hydrophobicity, antioxidant and antimicrobial activities

DS: Degree of substitution; Th: Thickness, mm; MC: Moisture content; WS: Water solubility; TS: Tensile strength, MPa; EB: Elongation at break; WVP: Water vapor permeability, g·m^−1^·s^−1^·Pa^−1^; WVTR: Water vapor transmission rate, g·h^−1^·m^−2^; OP: Oxygen permeability, cm^3^·m^−1^·d^−1^·Pa^−1^; SWCA: Static water contact angle; EC_50_: Antioxidant value against the DPPH radical (namely, the mass concentration of antioxidants produced a 50% scavenging effect against active free radicals), mg/mL; DPPH value: 2,2-diphenyl-1-picrylhydrazyl radical scavenging activity; E: Young’s modulus.

**Table 4:** Applications of five kinds of polysaccharide-based edible materials in food packaging.

Food		Edible Packaging & Preparation Method	St	Mass Loss/%	Dp/%	TSS/%	TA/%	pH	Vc Mass	TSP	Packaging Effects
Fruit	Strawberry	CMC/bacteriocin from *Bacillus methylotrophicus* BM47 coating; Dip-coating [27]	4–16 °C	10.5; 12 d	0; 12 d	8.6; 12 d	1.09; 12 d	3.34; 12 d	24.5; 12 d	9–10; 12 d	Reduce the weight loss and decay percentage of strawberries Inhibit the decrease of antioxidant activity and the propagation of the fungus Extend the shelf life by 4 or more days
		KGM/pullulan film; Casting [170]	4–14 °C	25		6	0.55		0.015 μg/mL		Decrease weight loss; Slow down fruit aging When the concentration of KGM was 1% with the mass ratio of KGM/pullulan 2:1, films exhibited the best preservation effect Extend the shelf life to 14 days
		CS/gelatin/thymol coating; Dip-coating [143]	4–7 °C	1–2	1.67	7.16				6.71	Both coatings protect strawberries against fungal (Botrytis cinerea) decay, improve the physiochemical parameters and shelf lives (extend by 2–3 days) The composite coating containing starch possesses higher TSP, antioxidant activity and catalase activity, and lower mass loss, Dp, TSS, guaiacol peroxidase, polyphenol oxidase, total anthocyanins, polygalacturonase and pectin-lyase than that containing gelatin; especially the antioxidant activity value/(mmoleq ascorbic acid/g food) of the former (26.34) was higher than the latter (25.31) The preservation effect of CS/starch/thymol coating was better than that of CS/gelatin/thymol coating
		CS/starch/thymol coating; Dip-coating [143]	4–7 °C	0.61	0	6.95				7.06	
	Grape	CS/*Mentha* (piperita L. or x villosa Huds) essential oil coating; Dip-coating [171]	25–12 °C; 12–24 °C			11.2–12.6° Brix	42.9–47.3 mmol H^+^/100 g food				Delay and even inhibit the appearance of postharvest mold (e.g., *Aspergillus niger, Botrytis cinerea, Penicillium expansum, and Rhizopus stolonifer*) infection in table grapes Reduce respiration and transpiration across the fruit surface, thus delaying senescence and extend shelf lives
	Banana	Rice starch/ι-carrageenan/sucrose fatty acid esters coating; Spray-coating [172]	20–14 °C	4.5		20.5°Brix	0.25				Reduce the weight loss, firmness (6.89 N), chlorophyll degradation, and respiration rate of Cavendish banana Delay the ethylene production and starch degradation rate during storage Extend the postharvest life for 12 days (40% extension) in the absence of refrigerated storage
	Guava	Acetylated cassava starch/hydroxyethyl cellulose coating; Dip-coating [96]	25–13 °C	13.15		8.0	0.66		20.5		Allow the guava respiration but still delayed the ripening process; Reduce mass loss, increase firmness, and maintain green skin color Extend the shelf life of guava
	Apricot	Basil seed gum/*Origanum vulgare* subsp. *viride* essential oil coating; Dip-coating [152]	4–8 °C	6.9		15				230	Kept quality and increased shelf-life of cut apricots Good antioxidant (EC_50_: 31.2 μg/mL; DPPH value: 22.7 g/kg) and antimicrobial properties (e.g., aerobic mesophilic, yeasts, and molds)
Vegetable	Cherry tomato	KGM/nisin coating; Spread-coating [173]	25–16 °C	9.5	Decay index: 0.133	6.22					Reduce the rotting index, weight loss rate, soluble solids content, and hardness of cherry tomato (Firmness of coated fruit was 47.02% higher than that of the control group) Induce peroxidase activity of cherry tomato Maintain sensory quality and extend shelf life
	Cucumber	Konjac glucomannan/saffron petal extract coating; Spread-coating [174]	4–5 °C			17.56				0.17	Reduce mesophilic bacteria and fungi populations; especially when the concentration of the extracts was 4%, the antimicrobial effect was most effective Improve the soluble solids, antioxidant activity, and soluble phenols (DPPH value could reach 20 mg/g) Decrease spoilage Keep quality features and prolong the shelf life
	Tomato/Chilly/Brinjal	CS nanoparticles coating; Dip-coating [56]	25–5 °C	0.21/ 3.3/ 0.53							Inhibit the growth of *Rhizoctonia solani, Fusarium oxysporum, Collectotrichum acutatum, and Phytophthora infestans* during storage Significant antioxidant activity; reduce the weight loss of vegetables, and prolong the shelf lives
**Food**		**Edible Packaging &** **Preparation Method**	**St**	**PV**	**TBARS**	**TVB-N**	**DPPH/%**	**ABTS/%**	**pH**	**TSP**	**Packaging Effects**
Nut	Pistachio	CMC/gelatin/*Dianthus barbatus* essential oil coating; Dip-coating [93]	25 °C–6 months	0.1–3.5							Slow down the lipid oxidation of pistachios Inhibit the growth of three aflatoxin-producing molds on pistachios, including *Aspergillus flavus* (PTCC-5004), *Aspergillus parasiticus* (PTCC-5286), and *Aspergillus parasiticus* (PTCC-5018) during storage Extend the shelf life
	Cashew nut	CS/mango leaf extract film; Casting [157]	30–28 °C	2.88							Better oxidation resistance than the commercial PA/PE and pure chitosan films Inhibit the lipid oxidation and remain the sensory quality of cashew nuts during storage
Meat	Chicken breast	Rye starch/Rosehip extract film; Casting [175]	4–9 °C		0.59		80.22	96.87			Reduce the generations of peroxide and TBARS; DPPH value of films was 25.62 mg GAE/g films Inhibit the lipid oxidation in chicken breast Prolong the shelf life
		Corn starch/gelatin/N-α-lauroyl-l-arginine ethyl ester monohydrochloride film; Casting [155]	4–19 °C		0.2				5.88		Good antimicrobial activity (The microbiological limit of acceptability for total viable counts was reached after 16 d) Composite films with non-oxidized starch better preserved the quality attributes of chicken than oxidized starch-based coating Extended the shelf-life of chicken to 16 d
		Oxidized corn starch/gelatin/N-α-lauroyl-l-arginine ethyl ester monohydrochloride coating; Spread-coating [155]	4–19 °C		1.52; 9 d				5.72; 9 d		Oxidized starch increased the antibacterial effectiveness, but enhanced lipid oxidation (the limit of acceptability in terms of TBARS was reached after 9 d) Extended the shelf-life of chicken to 9 d
	Pork	Cassava starch/*Lycium ruthenicum* Murr anthocyanins film; Casting [176]	25 °C–48 h			10.89–16 h; 17.21–24 h				6.15 −16 h; 6.49 −24 h	Delay the lipid oxidation of pork Achieve real-time and visual monitor for the pork freshness
	Beef loin	CS/cumin essential oil-loaded nanoemulsion film; Casting [177]	3–21 °C		1.39	12			5.4		Withstand low-dose gamma irradiation (GI) at 2.5 kGy Inhibited the growth of *L. monocytogenes*, *E. coli* O157:H7 and *Salmonella typhimurium* Slow down the increasing of TVB-N and pH Shelf life was extended at least 14 days combined with GI and refrigerated storage
	Ham	Iota-carrageenan/rosemary extract coating; Dip-coating [178]	5–15 °C								Inhibit the growth of aerobic mesophilic microorganisms, coliforms, lactic acid bacteria, and yeasts Remain the moisture, hardness (3779 g), and color of hams over the 15/days of storage
	Goat meat sausage	Maltodextrin/calcium alginate/*Tinospora cordifolia* extracts film; Casting [179]	−18–21 °C		0.54				6.79		Reduce the production of TBARS and free fatty acid (FFA); FFA reached 0.352% Oleic acid at 21 d Inhibit the reproduction of microorganisms (total plate, psychrophilic, yeast, and mold) Maintain the sensory quality of goat meat sausages
Oil	Olive oil	HPMC/cypress seed extract film; Casting [98]	23–23 °C	<20 (legal limit)							Slow down the oxidation of olive oil during 23 days of accelerated storage Shelf life could reach at least 7 days
	Soybean oil	Pomelo peel flours/tea polyphenol film; Casting [64]	23–15 °C	31.58			74.39				Significantly decrease peroxide value to delay oil oxidation during storage Inhibit the growth of *E. coli*, *S. aureus* and other bacteria, especially the inhibition of Gram-positive bacteria is stronger than Gram-negative bacteria
		Lime peel pectin/coconut water/lime peel extract film; Casting [180]	27–30 °C		3.39						Total phenolic content, DPPH value and ABTS value of composite films were 81.01 mg GAE/g film, 43.50 μM Trolox/g film, and 543.14 μM Trolox/g film, respectively Retarded soybean oil oxidation during storage by delaying hydroperoxide (primary lipid oxidation products) degradation
Aquatic product	Salmon	Cowpea starch/maqui berry extract film; Casting [181]	4–6 °C	1	0.63		42.39	88.46			Delay the lipid oxidation of salmon and extend its shelf life
	Hake	Agar/green tea extract/probiotic bacteria film; Casting [169]	4–15 °C			25			7.01		Inhibit the growth of spoilage microorganisms, especially H_2_S-producing bacteria Decrease the TVB-N, TMA-N, and pH value Increased the beneficial lactic acid bacteria in hake Extend the shelf life of hake at least for a week
	Flounder fillets	Agar/fish protein hydrolysate film; Casting [153]	5–15 °C			29.80			7.05		Decrease TVB-N and pH values; Delay the growth of bacteria groups, especially H_2_S-producing microorganisms Extend the shelf life of flounder fillets from 10 days to 15 days by improving biochemical and microbiological parameters in the last stages of the chilled storage Film with protein hydrolysate had no sensory limitation of essential oil, but its preservation effect was slightly lower
		Agar/clove essential oil film; Casting [153]	5–15 °C			25.83			6.76		
	Beluga sturgeon fillets	Jujube gum/nettle oil-loaded nanoemulsions coating; Coating [182]	4–15 °C	2.64	1.22	16.42 mg N/100 g			6.42		Warner–Bratzler shear force: 18.74 N; FAA: 0.94 Reduce the weight and cooking losses, and pH changes; Delay the textural and color deterioration Inhibit the lipid oxidation and foodborne bacteria growth; Prolong the shelf life
	Shrimp	Sweet potato starch/thyme essential oil coating; Dip-coating [183]	4–8 °C		0.3–0.5					8	Maintain the sensory properties (e.g., textural, hardness and color) and freshness Reduce pH value, lipid oxidation, bacteria count, and melanosis; Extend the shelf life

Note: St: Storage time, day; Dp: Decay percentage; TSS: Total soluble solids; TA: Titratable acidity; TSP: Total soluble phenolic, mg Gallic acid equivalent (GAE)/100 g food; Vitamin C mass: Vc mass, mg/100 g food; PV: Peroxide value, meq (peroxides or O_2_)/kg food; TBARS: Thiobarbituric acid reactive substances, mg malondialdehyde (MDA)/kg food. TVB-N: Total volatile basic nitrogen, mg/100 g food; TMA-N: Trimethylamine nitrogen, mg/100 g food; ABTS value: 2,2′-azino-bis (3-ethylbenzothiazoline-6-sulphonic acid) radical scavenging activity; DPPH value: 2,2-diphenyl-1-picrylhydrazyl radical scavenging activity.

**Table 5:** Dietary intake of acrylamide consumption [ng·(kg·body·weight)^−1^·day^−1^] based on the median of the estimated consumption of fried potatoes treated with the coating solutions. (Adapted with permission from Al-Asmar [199]; published by MDPI, 2018).

Age Groups	Control	Chitosan Coating	Pectin Coating
Toddlers	1387	858	720
Other children	1521	941	790
Adolescents	1072	663	557
Adults	719	445	374
Elderly	536	332	279
Very elderly	417	258	217

## Data Availability

Not applicable.
